# Endosomal Recycling Defects and Neurodevelopmental Disorders

**DOI:** 10.3390/cells11010148

**Published:** 2022-01-03

**Authors:** Shinji Saitoh

**Affiliations:** Department of Pediatrics and Neonatology, Graduate School of Medical Sciences, Nagoya City University, Kawasumi 1, Mizuho-cho, Mizuho-ku, Nagoya 467-8601, Japan; ss11@med.nagoya-cu.ac.jp; Tel.: +81-52-853-8246

**Keywords:** endosomal recycling, retromer, retriever, Schaaf–Yang syndrome, Ritscher–Schinzel syndrome

## Abstract

The quality and quantity of membrane proteins are precisely and dynamically maintained through an endosomal recycling process. This endosomal recycling is executed by two protein complexes: retromer and recently identified retriever. Defects in the function of retromer or retriever cause dysregulation of many membrane proteins and result in several human disorders, including neurodegenerative disorders such as Alzheimer’s disease and Parkinson’s disease. Recently, neurodevelopmental disorders caused by pathogenic variants in genes associated with retriever were identified. This review focuses on the two recycling complexes and discuss their biological and developmental roles and the consequences of defects in endosomal recycling, especially in the nervous system. We also discuss future perspectives of a possible relationship of the dysfunction of retromer and retriever with neurodevelopmental disorders.

## 1. Overview of the Endosomal Recycling System

**Figure 1:**
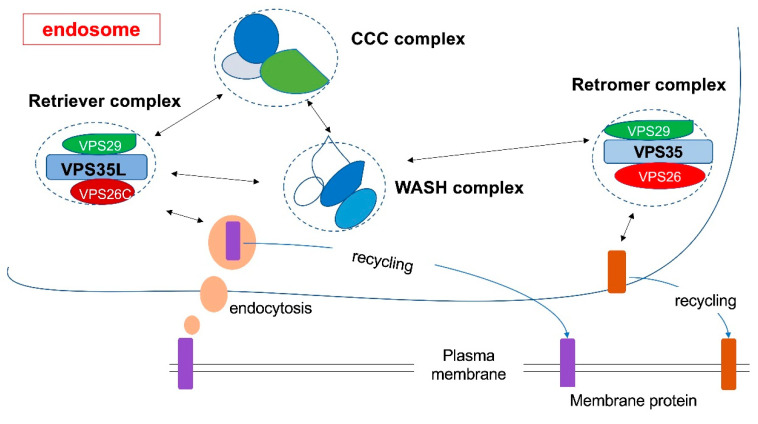
Schematic view of the endosome recycling system. Retromer and retriever complexes play a fundamental role in the recycling of numerous membrane proteins, although their target proteins are distinct. Retromer and retriever complexes interact with the WASH complex and the CCC complex for endosomal recycling (see text). WASH complex has an endosomal actin-remodeling function and is required for retromer and retriever to localize to the recycling endosome, while the CCC complex is shown to interact only with retriever.

Membrane proteins play a fundamental role in various cellular functions, including cellular adhesion, nutrient uptake, and signal transduction [1]. The quality and quantity of these proteins are precisely and dynamically maintained through a recycling process [2]. Membrane proteins are constantly internalized through endocytosis and transferred to an early endosome. The fate of the early endosome varies—through endosomal trafficking, it may be recycled to the cell membrane or degraded in lysosomes (Figure 1). The endosomal recycling system is thus crucial for maintaining functional membrane proteins. Defects in endosomal recycling have significant effects on cellular function. For example, the regular exchange of membrane proteins is essential for maintaining synaptic plasticity in neurons [3]. Therefore, defects in endosomal recycling could cause human disorders, including neurological diseases.

Two protein complexes, retromer and recently identified retriever, are involved in recycling pathways [4,5]. In this review, we discuss the role of these two recycling complexes and the consequences of defects in endosomal recycling. Recent identification of neurodevelopmental disorders caused by pathogenic variants in genes associated with retriever has expanded the significance of endosomal recycling in neurodevelopmental disorders as well as neurodegenerative disorders. Thus, we discuss future perspectives of a possible relationship of the dysfunction of retromer and retriever to neurodevelopmental disorders.

## 2. Retromer Complex

Retromer was originally identified in yeast as a complex required for retrograde trafficking of vacuolar cargo [6]. Retromer recycles cargo to both the *trans*-Golgi network (TGN) and the plasma membrane in all eukaryotes. It is a heterotrimer complex composed of three subunits: VPS35, VPS29, and VPS26 [7]. Two paralogues, VPS26A and VPS26B, are expressed in humans and play distinct roles. VPS35 binds to VPS26 at the N-terminal and to VPS29 at the C-terminal. Retromer is enriched on the cytosolic face of the early and late endosome. It uses various sorting nexin (SNX) proteins as cargo adaptors. Binding to SNX3 mediates the localization of retromer in the early endosome, while RAS-related protein RAB7-GTP localizes retromer in the late endosome. Sequence-specific cargo recognition of retromer is partly mediated by SNX27 [8]. SNX27 recognizes ΦxNPxpY or ΦxNxxpY as a sorting motif (where Φ is a hydrophobic residue and x is any residue) at the carboxy-terminal FERM-like domain. SNX27 also binds to a PDZ domain-binding sorting motif at the amino-terminal PDZ domain. More than 400 cargo proteins require SNX27 and retromer for their recycling [9,10]. Such cargo includes receptors and transporters that are essential for signal transduction, synaptic plasticity, and nutrient uptake.

## 3. Retriever Complex

Recently, a second retromer-like protein complex involved in endosomal recycling was identified and named retriever [5]. The retriever also forms a heterotrimer complex, and subunits are composed by VPS35L, VPS29, and VPS26C [4]. The structures of retromer and retriever are believed to be similar, although the precise structure of the retriever has not been determined. The retriever associates with SNX17, which recognizes ΦxNPxY or ΦxNxxY as a sorting motif [11]. SNX17 lacks the PDZ domain and thus does not associate with the proteins containing the PDZ-binding motif. The retriever binds to the COMMD/CCDC22/CCDC93 (CCC) complex and forms the commander complex, which is implicated in the trafficking of a variety of membrane proteins [12]. The commander complex interacts with the Wiskott–Aldrich Syndrome Protein and SCAR Homolog (WASH) complex, which has an endosomal actin-remodeling function, and with SNX17. The WASH complex also interacts with retromer for endosomal recycling, although the commander complex is thought to interact only with retriever [4]. Target proteins of the commander complex include the ATP7A/ATP7B copper transporter, low-density lipoprotein receptor (LDLR) family proteins, and α5β1 integrin [12].

## 4. Relationship between Retromer and Retriever

Thus far, retromer and retriever are the only complexes proven to be involved in membrane protein recycling. Because of the architectural similarity, both complexes are thought to have evolved to play overlapping roles in membrane protein recycling. Indeed, VPS29 is shared in both complexes. The expression patterns of retromer and retriever in tissues, including brain, mostly overlap, although information is limited. Therefore, distinct roles of retromer and retriever appear to be determined by their target proteins. The specificity of target proteins is determined by adaptor proteins SNX27 and SNX17, which bind to VPS35 and VPS35L, respectively. As described above, SNX27 recognizes ΦxNPxpY or ΦxNxxpY as a sorting motif, while SNX17 recognizes ΦxNPxY or ΦxNxxY. Therefore, retromer and retriever complexes recycle different membrane proteins based on the sequences of the sorting motif. For example, αβ integrin is only recycled by retriever but not by retromer [5]. Nevertheless, since many membrane proteins interact, a dysfunction of retromer or retriever could affect overlapping proteins directly or indirectly [5]. Revealing the functional interactions of retromer and retriever complexes in future studies is of great interest.

## 5. Human Disorders Associated with Retromer Dysfunction

**Figure 2:**
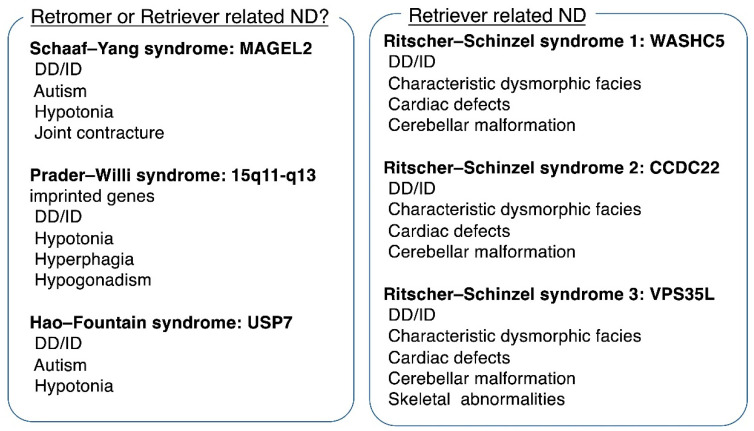
Putative neurodevelopmental disorders associated with retromer and retriever complexes. Major clinical features are demonstrated for each disorder. ND, neurodevelopmental disorder; DD, developmental delay; ID, intellectual disability.

Retromer dysfunction is involved in several human diseases, in particular, neurodegenerative disorders including Alzheimer´s disease (AD) and Parkinson´s disease (PD) (Figure 2). Since endosomal recycling is important in synaptic plasticity and maintenance of neuronal health, neurons are highly vulnerable to a dysfunction of endosomal recycling.

The link between AD and retromer was first found in 2005. Using postmortem brains from AD patients and controls, Small et al. discovered that two retromer subunits, VPS26 and VPS35, were deficient in brains from patients with AD [13]. Subsequently, a line of genetic studies revealed the link between AD and retromer-related genes. Examples include *SORL1*, *SNX1*, *SNX3*, and *RAB7A* [14]. As described above, *SNX3* and *RAB7A* encode major proteins binding to retromer.

The link between PD and retromer was first demonstrated by whole-exome sequencing. In 2011, autosomal dominant mutations in *VPS35* were identified in patients with late-onset PD [15,16]. Subsequent studies revealed that the mutations caused a reduction in VPS35 binding to the WASH complex, and thereby induced α-synuclein aggregates, which have a pathogenic role in PD, although non-neuronal cells were used for the experiments [17,18]. It is of interest that most risk or causal genes of PD are involved in autophagy–lysosomal–endosomal pathways. Dysfunction of these pathways therefore might induce an accumulation of α-synuclein, which could be associated with the development of PD. However, the precise pathophysiology of *VPS35* mutations in PD remains to be elucidated, and further investigation is necessary.

A dysfunction of retromer is also suggested in Down syndrome (DS) [19]. Individuals with DS have an increased risk of developing AD: more than 60% of individuals with DS will show clinical features of AD by 40 to 50 years of age [20]. Importantly, the neuropathological findings in DS are in line with those of AD, as accumulating amyloid-β (Aβ) is found in elder individuals with DS who show cognitive decline as well as in patients with AD. This might be related to the fact that the Aβ precursor protein (APP) gene is located on chromosome 21. Aβ is first accumulated in the endosome system in individuals with DS as well as in those with AD, indicating that endosomal trafficking including retromer is likely to play an important role in the clearance of Aβ [21]. Recently, dysregulation of the retromer complex was reported in postmortem brain tissues and fibroblasts derived from individuals with DS [22]. Although the cause of dysregulation of the retromer complex in DS remains to be uncovered, dysfunctional endosomal recycling may play a significant role in the development of AD in individuals with DS.

Although the link between retromer dysfunction and AD, PD, or DS has been intensively studied, a pathogenic role for retromer in neurodevelopmental disorders remains to be uncovered. Although it is yet to be investigated, a putative example could be Schaaf–Yang syndrome (SYS). SYS is a congenital disorder characterized by developmental delay, autism spectrum disorder, and multiple congenital joint contractures [23,24]. SYS is known to resemble Prader–Willi syndrome (PWS), which is a distinct developmental disease, particularly beyond infancy [25]. SYS is caused by a mutation in *MAGEL2*, which is a maternally imprinted gene located in the 15q11-q13 chromosome region [23]. *MAGEL2* encodes a protein expressed predominantly in the brain. MAGEL2 protein binds the E3 RING ubiquitin ligase TRIM27 as well as USP7, which is a deubiquitinating enzyme and is recruited to the endosome through interaction with the WASH complex [26]. This complex is named the MUST (MAGEL2-USP7-TRIM27) complex. The MUST complex may facilitate the retromer endosomal recycling pathway through ubiquitination and activation of the WASH complex, which interacts with retromer as well as with retriever [4]. Therefore, *MAGEL2* mutations reduce the function of the MUST complex and subsequently perturb endosomal recycling, which may underlie the pathophysiology of SYS. Some patients with SYS show neurological regression resembling encephalopathy after febrile infectious diseases [25]. Since retromer dysfunction is related to neurodegenerative disorders, it is of interest whether this acute neurological dysfunction in patients with SYS in febrile conditions is related to endosomal recycling dysfunction. Under stress conditions, including febrile episodes, neurons may need to adopt a rapid turnover of membrane proteins. Therefore, partial dysfunction of endosomal recycling might manifest as neurological dysfunction under critical conditions only. Therefore, not only neurodegeneration but intermittent neurological dysfunction under certain conditions may be a phenotype associated with endosomal recycling dysfunction.

Mutations identified in SYS patients are exclusively truncating mutations [23,24,25]. *MAGEL2* is an intronless gene, and thus it escapes nonsense-mediated mRNA decay. Therefore, accumulation of truncated protein is expected. In contrast, a deletion of the entire *MAGEL2* gene of paternal origin is associated with milder phenotypes presenting as developmental delay but without joint contractures [27,28]. *MAGEL2* is only expressed from the paternally derived allele and is located in the common deletion region of PWS. PWS is caused by a paternal deletion of approximately 4Mb in the 15q11-q13 region, which includes *MAGEL2* [29]. Therefore, a PWS patient with a common deletion also lacks paternally derived *MAGEL2*. PWS patients show developmental delay/intellectual disability (DD/ID), neonatal hypotonia, hyperphagia resulting in severe obesity, and hypogonadism [29]. A patient with a deletion of the entire *MAGEL2* shows DD/ID and autism but no hyperphagia or hypogonadism [27,28]. Therefore, haploinsufficiency of *MAGEL2* should partially contribute to the development of DD/ID in PWS patients. Thus, PWS could be categorized as one of the endosomal recycling-related neurodevelopmental disorders. Nevertheless, the phenotype of SYS, including intellectual disability and joint contracture, is more severe than that of PWS. Collectively, *MAGEL2* mutations in SYS patients should disturb the function of the MUST complex more intensively than in PWS, in which haploinsufficiency of *MAGEL2* may cause mild dysfunction of the MUST complex.

Mutations in *USP7* were also identified in patients with developmental delay, altered behavior, and neurologic anomalies including white matter changes [30,31]. Fountain et al. comprehensively described the clinical features of 23 patients [31]. Most patients with a *USP7* mutation showed DD/ID of variable range, autism, and hypotonia, whereas dysmorphic features were not conclusive. It is of note that patients with a *USP7* mutation showed brain abnormalities associated with white matter volume loss and thinning of the corpus callosum, although gross brain anomalies were not seen. Recently, USP7-related neurodevelopmental disorder was named Hao–Fountain syndrome (HFS). Mutated USP7 was shown to lack activation of the WASH complex and thereby decreased membrane protein levels including M6PR [30]. It is of interest that patients with a mutation in *USP7* show phenotypes (intellectual disability, autism, and hypotonia) overlapping with SYS and PWS, suggesting that these disorders share pathological mechanisms in brain [32].

Dysfunction of the MUST complex is likely to cause neurodevelopmental disorders including SYS, PWS, and HFS. The consequence of dysfunction of the MUST complex might be perturbation of the recycling of membrane proteins. Nevertheless, the link between MUST complex and endosomal recycling still remains to be investigated. Moreover, it is not clear whether retromer or retriever is involved because the WASH complex interacts with both retromer and retriever. Nevertheless, as described in the following section, neurodevelopmental disorders caused by dysfunction of retriever tend to show distinct clinical features. Thus, the involvement of retromer in disorders with a dysfunction of the MUST complex deserves to be investigated.

## 6. Mouse Models of Retromer Function

Several mouse models have been established to study retromer function. Homozygous deletion of core subunits of retromer in mice cause lethal phenotypes during embryonic stages [33], suggesting a fundamental significance of retromer. Hemizygous deletion of *Vps35* in mice show almost normal phenotypes; however, when these mice are crossed with AD model mice, the resulting double heterozygous mice show earlier development of AD phenotypes including accumulation of Aβ [33]. Therefore, a causal relationship of retromer dysfunction and AD development was suggested in mice studies.

For the relationship between *VPS35* and PD, *VPS35* D620N knock-in (KI) mice were produced and both heterozygous and homozygous *VPS35* D620N mice presented clinical features of PD in an age-dependent manner [34]. *VPS35* D620N KI mice showed a decrease in dopamine neurons [34,35]. Unexpectedly, *VPS35* D620N KI mice showed robust tau-positive neurons, which is related to AD, but lacked the accumulation of α-synuclein that is expected for PD. Heterozygous *Vps35* null mice or conditional *VPS35* KO mice in dopamine neurons did not show the accumulation of tau but presented several features of PD [36,37]. Therefore, loss of function of VPS35 in dopamine neurons induces neurodegeneration and underlies the neuropathology of PD. It is of note that the *VPS35* D620N mutation is suggested to have a gain-of-function mechanism [36]. Therefore, precise control of membrane proteins by retromer complexes, at least in dopamine neurons, is essential to prevent neurodegeneration, which in turn causes PD.

Two lines of *Magel2* knock-out (KO) mouse models have been established [38,39]. One mouse model, which lacked most of the *Magel2* gene and its promoter, showed hypothalamic dysfunction, decreased pre-wean weight gain, circadian dysfunction, altered appreciation of novelty, and a mildly increased neonatal lethality [38]. The other mouse model showed phenotypes of disturbed sociability, decreased suckling, decreased oxytocin levels, and approximately 50% of neonatal lethality [39]. Therefore, the phenotypes of the two mouse models overlapped but were not identical. Therefore, we have introduced a frameshift mutation in *Magel2* and created SYS model mice because patients with SYS carry a truncating mutation in paternally derived *MAGLE2* [40]. *Magel2* is a maternally imprinted gene, and SYS model mice maintained proper imprinting and expression patterns in the brain. Unexpectedly, SYS model mice showed very mild phenotypes compared with those of previously created mouse models and human phenotypes. Ongoing studies of retromer function in the SYS mouse model would reveal the significance of the MUST complex in retromer function.

## 7. Human Disorders Associated with Retriever Dysfunction

Unlike with retromer dysfunction, in patients with a pathogenic mutation in genes associated with retriever function, congenital diseases have been identified. Thus far all mutations are found in patients with similar phenotypes, called Ritscher–Schinzel syndrome (RSS) (Figure 2). RSS is a developmental malformation syndrome characterized by craniofacial abnormalities, congenital heart defects, and cerebellar brain malformation [41]. RSS is also called 3C syndrome representing the craniofacial, cardiac, and cerebellar phenotype although not all features are necessarily present. In 2013, a homozygous mutation in *KIAA0196* was first identified in patients with RSS [42]. Elliott et al. reported a large pedigree of ten affected patients with a homozygous splicing mutation in *WASHC5*. All patients had DD/ID and dysmorphic facies including a prominent forehead, low posterior hairline, wide and downward-slanting palpebral fissures, hypertelorism, and low-set ears. Approximately half of the patients showed cardiac anomalies and brain anomalies including Dandy–Walker malformation and hypoplasia of the cerebellar vermis. Therefore, cardiac or brain anomalies are not present in all patients. *KIAA0196* was renamed *WASHC5*, which encodes strumpellin, a subunit of WASH complex. RSS with a mutation in *WASHC5* is called RSS-1.

In 2015, a hemizygous missense mutation in *CCDC22* located in Xp11.23 was identified in patients with RSS phenotypes [43]. CCDC22 is a component of the CCC complex, which interacts with retriever. Kolanczyk et al. reported two male siblings who showed DD/ID, facial dysmorphism, cardiac anomaly (ventricular septum defect), and cerebellar malformation (Dandy–Walker malformation). Their facial features included upward slanting palpebral fissures, wide-set eyes, short philtrum, protruding tongue, and a broad neck, making their facial appearance similar to those of RSS1 patients. RSS with a mutation in *CCDC22* is named RSS-2.

Lastly, in 2020, Kato et al. reported siblings showing RSS-like phenotypes associated with compound heterozygous mutations in *VPS35L*, a core subunit of retriever [44]. The siblings presented with DD/ID, craniofacial dysmorphism including prominent forehead, arched eyebrows, downward-slanting palpebral fissures, upturned nose, thin upper lip, and micrognathia. Both siblings had cerebellar malformation (vermis hypoplasia), and one sibling had a cardiac defect (atrioventricular septum defect). Notably, the siblings showed skeletal abnormalities including chondrodysplasia punctata. Their craniofacial dysmorphisms were consistent with those of RSS1 and RSS2, indicating that characteristic craniofacial dysmorphism is specific for RSS. Functional studies revealed that the mutated VPS35L failed to bind to VPS29 and subsequently degraded, clearly leading to a loss of function of the retriever complex. RSS with a mutation in *VPS35L* is named RSS-3.

Recently, Jeanne et al. reported nine patients, including one patient from the first family reported by Ritscher et al., and identified missense variants in *DPYSL5* [45,46]. A recurrent de novo p.Glu41Lys was found in eight unrelated patients, while a p.Gly47Arg was identified in one patient from the original RSS family. DPYSL5 belongs to the collapsing response mediator protein family and an intracellular mediator of neurotrophic factors regulating neurite structure formation. Therefore, a direct link between DPYSL5 and retriever has not been demonstrated.

Collectively, a deficiency of the retriever–CCC–WASH complex causes neurodevelopmental disorders represented by RSS. Common features of RSS are developmental delay or intellectual disability and characteristic facial features, although other malformations including cardiac defects are associated to varying degrees. Therefore, normal function of retriever endosomal recycling is crucial for brain and craniofacial development as well as for the development of other organs.

## 8. Mouse Models for Retriever Function

Owing to the recent identification of the retriever complex, limited information is available for retriever function in the mouse model. Thus, we introduced a truncating mutation in *Vps35l* and created model mice [44]. Homozygous *Vps35l* KO mice died before embryonic day 10.5, suggesting a fundamental role of retriever complex in fetal development. Heterozygous *Vps35l* showed normal phenotypes corresponding to the human phenotypes. Because of embryonic lethality, it is difficult to test the significance of the retriever complex in development. Thus, conditional KO mice would be of value to investigate the retriever function in development. Additionally, as in *Vps35* KO mice, crosses with AD model mice or other neurodegeneration model mice would be of interest.

## 9. Neurodevelopment and Endosomal Recycling

It is easily understood that proper homeostasis of membrane proteins including receptors and transporters is essential for brain development and function. Thus, defects in endosomal recycling are likely to affect brain development and function. Nevertheless, neurodevelopmental features associated with either retromer dysfunction or retriever dysfunction appear to be distinct, indicating different roles of each system in the brain.

Target membrane proteins of retromer include AMPA and NMDA receptors, which are essential for normal synaptic communications and brain function [9,10]. It is of note that compound heterozygous mutations in *SNX27*, which binds retromer, were identified in patients with epilepsy, developmental delay, and subcortical white matter abnormalities [47]. Patients with homozygous truncating mutations in *SNX27* showed more severe phenotypes of infantile myoclonic epilepsy, developmental delay, cardiac defects, and neurodegeneration of progressive neurological deficits [48]. These reports indicated a critical role of retromer in the developing brain, especially in excitatory–inhibitory balance.

Retriever target proteins are numerous among integral proteins required for cell adhesion, including numerous integrins [12]. Cell–cell interaction is crucial in brain development; thus, such difference in target proteins may partly explain the distinct role of retromer and retriever in brain development.

## 10. Perspectives of Endosomal Recycle Disorders

Endosomal recycling dysfunction is growingly recognized as a new entity of neurodevelopmental disorders. Nevertheless, few genes involved in the endosomal recycling system have been identified as causative genes for neurodevelopmental disorders, even though many genes are involved in endosomal recycling. In addition, if we expand our view to the endosomal trafficking system, more and more genes play significant roles. Thus, it is plausible that more genes in endosomal recycling await to be identified as novel causative genes. For the retriever system, the RSS phenotype would be a clue for the identification of such new genes.

For the retromer system, most genes identified in association with retromer are related to neurodegenerative disorders including AD or PD. SYS or USP7-related disease may be associated with a dysfunction of retromer, although evidence is limited. Given the fundamental roles of retromer in appropriate maintenance of membrane proteins, more genes related to the retromer recycling complex are likely to be associated with neurodevelopmental disorders. The phenotypes of retromer-related neurodevelopmental disorders might not be as specific as those related to retriever.

Endosomal recycling targets numerous membrane proteins, which seemingly complicates developing specific treatment for neurodevelopmental disorders associated with dysfunction of retromer or retriever. Catalogues for dysregulated genes for each disorder would be of value for the development of pathophysiology-based therapeutic interventions. Alternatively, improvement in endosomal recycling through upregulating retromer or retriever function would be a novel therapeutic target for certain neurodevelopmental or neurodegenerative disorders. Indeed, such attempts have been tried for AD. Li et al. introduced AAV-VPS35 into the brain of 3xTg mice, which are model mice developing AD-like neuropathology and behavioral deficits [49]. They found that VPS35 overexpression ameliorated behavioral deficits as well as neuropathology. Therefore, targeting endosomal recycling would be a new paradigm for identifying treatments for neurodevelopmental as well as neurodegenerative disorders.
